# Intraspecific Variability Largely Affects the Leaf Metabolomics Response to Isosmotic Macrocation Variations in Two Divergent Lettuce (*Lactuca sativa* L.) Varieties

**DOI:** 10.3390/plants10010091

**Published:** 2021-01-05

**Authors:** Giandomenico Corrado, Luigi Lucini, Begoña Miras-Moreno, Leilei Zhang, Christophe El-Nakhel, Giuseppe Colla, Youssef Rouphael

**Affiliations:** 1 Department of Agricultural Sciences, University of Naples Federico II, 80055 Portici, Italy; christophe.elnakhel@unina.it (C.E.-N.); youssef.rouphael@unina.it (Y.R.); 2 Department for Sustainable Food Process, Research Centre for Nutrigenomics and Proteomics, University Cattolica del Sacro Cuore, 29122 Piacenza, Italy; mariabegona.mirasmoreno@unicatt.it (B.M.-M.); leilei.zhang@unicatt.it (L.Z.); 3 Research Centre for Genomics and Bioinformatics (CREA-GB), via San Protaso 302, 29017 Fiorenzuola d’Arda, PC, Italy; 4 Department of Agriculture and Forest Sciences, University of Tuscia, 01100 Viterbo, Italy; giucolla@unitus.it

**Keywords:** macronutrients, nutrient availability, omics, adaptation, primary metabolism, secondary metabolism, leaf, breeding

## Abstract

Mineral elements are essential for plant growth and development and strongly affect crop yield and quality. To cope with an everchanging environment, plants have developed specific responses to combined nutrient variations. In this work, we investigated the effects of multifactorial treatments with three macrocations (K, Ca, and Mg) on lettuce (*Lactuca sativa* L.) varieties that strongly diverge in leaf pigmentation (full red or green). Specifically, we monitored main leaf parameters and metabolomics profiles of hydroponically grown plants fed with isosmotic nutrient solutions that have different proportions of macroelements. The result revealed a high biochemical plasticity of lettuce, significantly affected by the genotype, the nutrient solution, and their interaction. Our work also provided evidence and insights into the different intraspecific responses to multifactorial variation of macrocations, with two varieties having distinct strategies to metabolically respond to nutrient variation. Overall, plant adaptive mechanisms increased the phytochemical diversity between the varieties both among and within the main classes of plant secondary metabolites. Finally, our work also implies that the interaction of a pre-existing phytochemical diversity with the management of multiple mineral elements can offer added health-related benefits to the edible product specific to the variety.

## 1. Introduction

The availability of mineral elements is essential for any living organism and is of fundamental importance for crop production [1,2]. Considerable knowledge has been accumulated about the biological roles and metabolic mechanisms related to inorganic elements, especially those traditionally classified as the essential macronutrients for higher plants (i.e., N, P, K, Mg, Ca, and S) [3]. The effects on plants of each nutrient have mainly been studied individually [4]. Considering the complexity of the rhizosphere, these studies are typically carried out with plants growing with their roots in a water-based nutrient solution (NS) without soil (i.e., hydroponics), thus allowing researchers to experimentally control and modulate the pH, concentration, and composition of the NS [5]. It is well established that deficiency and excessive supply of any mineral element can produce morphological, biochemical, and metabolomic variations that significantly affect plant growth and physiology [2,6,7]. These studies have provided valuable information for both modern agriculture and environmental protection, however, it has become increasingly clear that this experimental approach does not fully replicate agronomic conditions, in which plants experience a simultaneous variation of different nutrients. In addition, mineral deficiency or oversupply rarely occur in professional horticulture. The effect of mineral bioavailability on plant metabolism is also complicated by significant interactions among chemical elements, with a range of positive and negative outcomes described in the literature [8,9]. There is growing evidence that nutrient interaction is a complex phenomenon that occurs both at the soil and plant level [10,11]. Although different abiotic factors and chemical characteristics are involved, mineral interaction is expected to be more common for elements that share similar chemical properties (e.g., size and charge), such as inorganic cations or anions [12].

Over the last decade, the advent of “omics” technologies has enabled a deeper understanding of the effects of several experimental factors in horticulture [13,14]. For instance, metabolomics has allowed researchers to unravel the complex, multifaceted response that plants adopt under stress conditions [15,16,17,18]. Metabolomic studies are also significant considering the ample intraspecific differences in the metabolic profiles that may exist within the germplasm of a crop [19,20,21]. Secondary metabolites represent the functional component of plant food and these compounds are expected to be highly responsive to mineral availability [22,23,24]. 

The supply of a balanced amount of nutrients is among the most important preharvest factors that directly influence crop yield and quality. Recent studies have indicated that the plant response to combined nutrient variations is something besides the responses to each individual element [4]. While different works have indicated the existence of a specific molecular crosstalk in nutrient sensing, and of ample homeostatic interactions in plants [4], relatively less is known on the large-scale metabolic alterations specifically related to combined variation of nutrient availability. Knowledge of this area is crucial in plant science to design more nutrient-efficient strategies for a sustainable increase in crop yield [25], and also as a powerful means to tailor food quality going beyond the single nutrient approach [26].

The aim of this work was to study the effect of concurrent variation in the availability of macrocations in lettuce varieties that differ in their profile of secondary metabolites. Lettuce (*Lactuca sativa* L.) is a commercially important leafy vegetable that is becoming the reference species for the Asteraceae, one of the largest and more diverse plant families [27]. This species comprises several horticultural groups that exhibit a strong morphological variation. In this work, we focused on two morphologically similar butterhead varieties that strongly diverge in leaf color, either green or (full) red. These two leaf colors typify those found in various lettuce horticultural types. In lettuce, as well as in many other plant species, differences in the amount and type of several phytochemicals are made evident by the color of the leaves [28,29]. Among macronutrients, we selected calcium, magnesium, and potassium because they are taken up by the roots almost exclusively as free cations [30]. Moreover, plants are the main dietary source of these essential elements [31]. Finally, these plant macronutrients, together with sodium and chloride, are mobile and present in plant cells in ionic form, as solutes in the cytosol and vacuoles, and associated with a plethora of carbon-containing compounds [30]. In this work, using a hydroponics cultivation system, we tested three different NSs, each having the dominance of one macrocation. In each solution, the concentrations of the analyzed cations were well within those expected to give rise to acute deficiency symptoms and inhibitory oversupply, easing possible knowledge translation into horticultural applications. To untangle the effect of mineral availability from that due to the osmolarity variation in the NS, we employed isosmotic nutrient solutions based on fixed ratios among macrocations. This also ensured an identical, optimal cations-to-anions ratio for plant physiology.

## 2. Results

**Figure 1:**
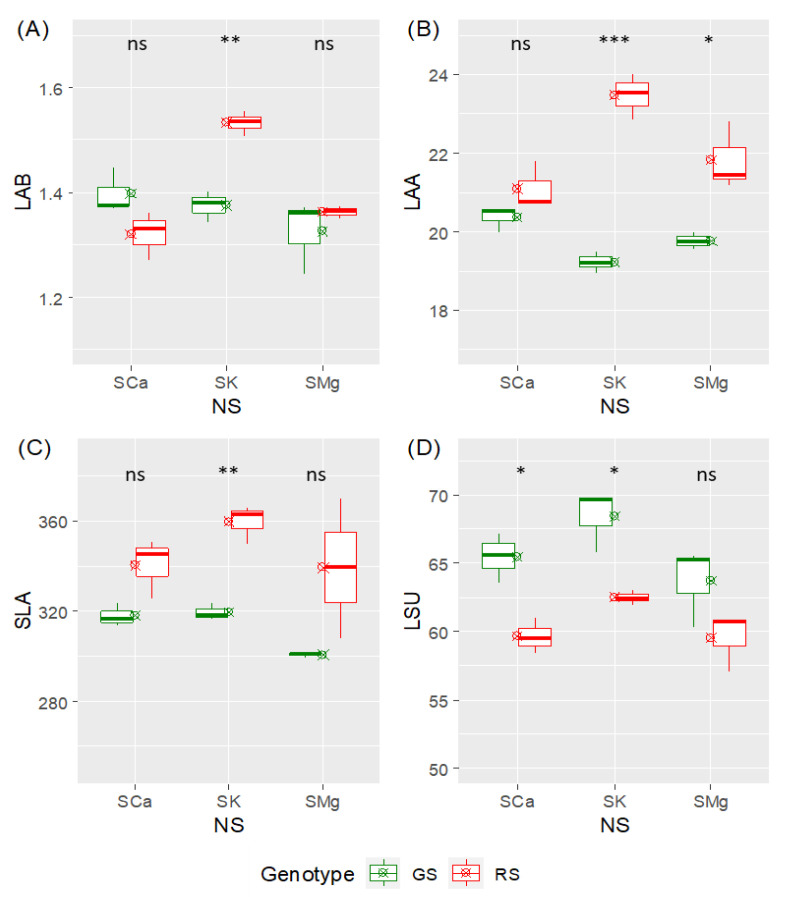
Box and whisker plots of the leaf morphological parameters. A crossed circle indicates the mean value. (**A**) LAB, leaf average fresh biomass (g); (**B**) LAA, leaf average area (cm^2^); (**C**) SLA, specific leaf area (cm^2^/g); (**D**) LSU, leaf succulence (mg/cm^2^). NS, nutrient solution; GS, ‘Green Salanova’; RS, ‘Red Salanova’). SCa, SK, and SMg, nutrient solutions with a dominance of calcium (Ca), potassium (K), and magnesium (Mg), respectively. For each pairwise comparison, asterisks denote a statistically significant difference (*** *p* < 0.001, ** *p* < 0.01, * *p* < 0.05, and ns, not significant) according to the Student’s independent *t*-test.

A previous morphological study indicated that the most significant effects of the altered macrocation ratio in differently colored lettuce varieties were recorded on leaf traits [32]. For this reason, in this work, we focused on leaf parameters to examine the influence of the two factors under investigation, namely, the variety (i.e., genotype, G) and the macrocation composition (i.e., the nutrient solution, NS). A two-way analysis of variance indicated that the leaf average fresh biomass (LAB) was not affected by the genotype, while the NS had a significant influence (Supplementary Table S1). Moreover, a clear interaction between the NS and the G was present. Specifically, a positive effect on the LAB of the ‘Green Salanova’ (as compared with ‘Red Salanova’) was recorded only for the nutrient solution with a dominance of potassium (SK) (Figure 1A).

Factor interaction was also present for the leaf average area (LAA) because, for instance, a positive effect for the ‘Red Salanova’ was present only for the SK solution and, to a lower extent, for the nutrient solution with a dominance of magnesium (SMg). LAA, differently from the LAB, was affected by the genotype, while the NS did not have a significant impact (Figure 1B). It is noteworthy that the specific leaf area (SLA), one of the leaf traits that better correlates the relative plant growth [33], was under a genotypic control, because overall the ‘Red Salanova’ displayed a larger carbon gain (in terms of biomass) relative to the leaf area (Supplementary Table S1), while the effect of the NS and factor interaction were not significant. The post hoc pairwise comparison indicated that a higher SLA was evident for the SK solution. Compared to the red genotype, the leaves of the ‘Green Salanova’ had a lower tissue density (e.g., they were more succulent) and this variable was strongly affected by the factors, G and NS (Supplementary Table S1). The post hoc analysis indicated that this difference was not significant only with the SMg solution. Overall, the data indicated that some key parameters that define the main morphological attributes of the lettuce leaves, in addition to yield [32], are differentially affected by the plant variety and the nutrient solution. Factor interaction was present for the leaf area and biomass. The largest differences between varieties were recorded for the SK solution for all parameters, pointing towards a specific different metabolic response of the varieties to the nutrient solutions. Moreover, the different macrocation ratios did not have an intraspecific effect on SLA and this, in non-limiting water and nutrient conditions, was compensated by the tissue density of the leaves.

### 2.1. Metabolomics Profiling

#### 2.1.1. The Genotype, the Nutrient Solution (NS) and Their Interaction Specifically Vary the Leaf Metabolome

**Figure 2:**
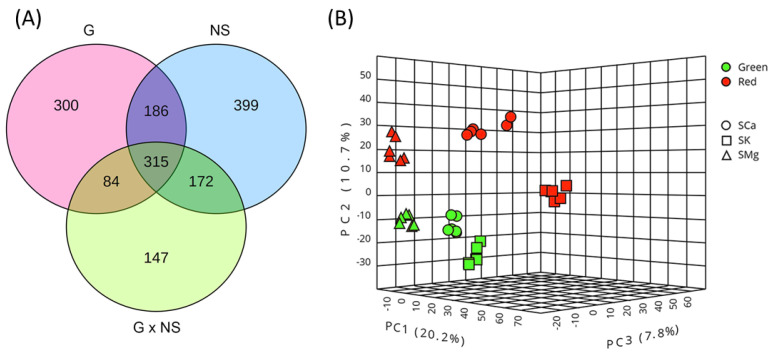
(**A**) Venn diagram of two-way ANOVA test for the leaf metabolites. The graph reports the number of common and factor-specific differentially accumulating compounds (*p* < 0.05) considering the factors, genotype (G), nutrient solution (NS) and their interaction (G × NS); (**B**) PCA three-dimensional (3D) score plot of the samples under investigation. For each sample, the color indicates the variety (green, ‘Green Salanova’ and red, ‘Red Salanova’), and the shape indicates the nutrient solution (circle, SCa; square, SK; and triangle, SMg).

To examine the influence on the leaf metabolome of the genotype and the nutrient solution, we first performed a two-way analysis of variance. Taking into account the absolute number of differentially accumulating metabolites, this analysis indicated that the NS had the larger effect (*n* = 1072), followed by the G (*n* = 885). Significant interactions were also evident (*n* = 718) yet, a relatively high number of compounds (*n* = 315, 42%) were affected by the genotype, the nutrient solution, and their interaction, as illustrated by the Venn diagram (Figure 2A).

**Figure 3:**
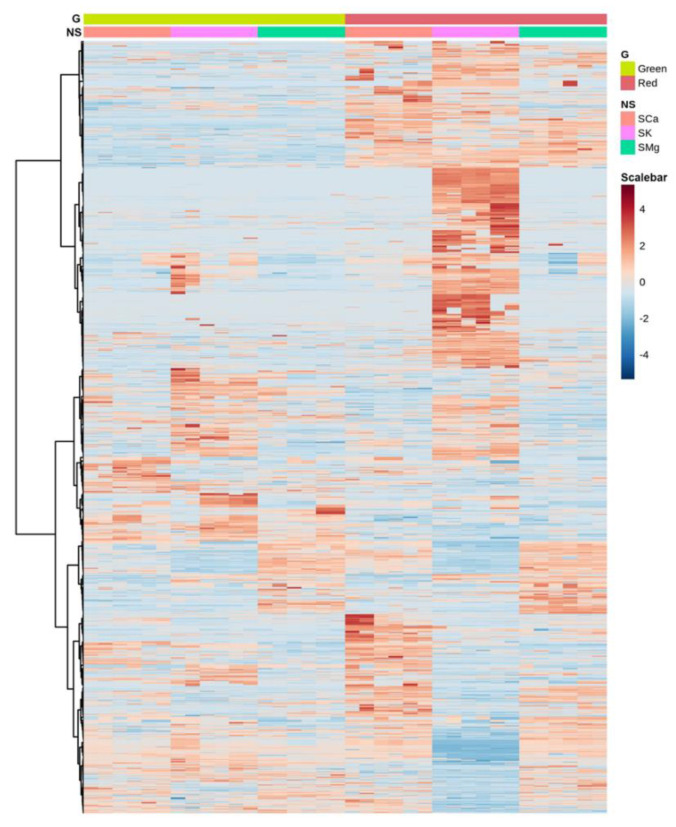
Heatmap clustering of the leaf metabolomes in the different experimental conditions. Individual samples are in columns. Samples are identified by the colored bars on the top. Clustering of the compounds was performed with Ward algorithm based on Euclidean distances.

Moreover, the PCA analysis showed that the biological replicates coherently clustered in distinct spaces, indicating that the impact of the experimental factors on the lettuce metabolism was substantial and specific (Figure 2B). To visualize the metabolic profiles of each condition, we generated a heatmap based on all the significantly different compounds (two-way ANOVA), which were hierarchically clustered (HC) considering the Ward algorithm based on Euclidean distances of their across-sample normalized quantity. This analysis illustrated that each experimental condition was largely characterized by a distinctive metabolic profile also in quantitative terms (Figure 3). For instance, the HC indicated the presence of groups of metabolites significantly increased in the ‘Red Salanova’ in all NSs, as well as clusters of compounds differentially expressed only for a specific nutrient solution.

**Figure 4:**
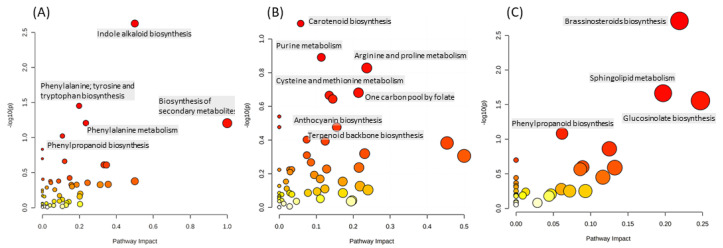
Metabolic pathway analysis of the compounds exclusively altered by the genotype (panel **A**), the nutrient solution (panel **B**), and the interaction of genotype and nutrient solution (panel **C**). The identified metabolic pathways are represented as circles, whose color and size are based, respectively, on the *p*-values, calculated with the hypergeometric test (from white to deep red) and on the pathway impact values, from the smallest to the largest. The non-quantitative pathway analysis used the Arabidopsis metabolome KEGG library set as reference.

To have an overview of the metabolomic changes induced by the two factors and their interaction, we carried out a biological interpretation of the metabolite set using a pathway-centered enrichment approach. To this aim, we considered compounds that were only affected by the genotype (*n*= 300), the nutrient solution (*n* = 399), or their interaction (*n* = 147). The enrichment analysis based on Kyoto Encyclopedia of Genes and Genomes (KEGG) codes indicated that the main effects of the factors under investigation are different (Figure 4). The variation in the metabolites significantly affected only by the genotype impacted pathways mostly related to the production of secondary metabolites such as (mono)terpenoids, and precursors and metabolites of the phenylpropanoid class. The factor NS significantly affected a more diverse set of pathways, which included both primary (mainly amino acids) and secondary metabolism such as carotenoids, anthocyanins, and the biosynthesis of the terpenoid backbone. In primary metabolism, different compounds related to a glutamate-deriving proline biosynthesis (e.g., hydroxyproline, L-proline, hydroxyglutamate semialdehyde, and glutamate 5-semialdehyde) were also affected. The NS impacts on pathways and their statistical significance were lower than the G factor, despite a higher number of differentially accumulating compounds. This may be because the different levels of the NS factor (i.e., the different macrocation ratios) have specific effects. This explanation is also consistent with the low number of metabolites that are exclusively affected by the G × NS interaction. The most impacted pathways by G × NS were the brassinosteroid biosynthesis (especially brassinolides’ precursors such as the 6-deoxocastasterone and the 6-deoxotyphasterol), and those related to the sphingolipids, and to compounds that are classified in Arabidopsis as precursors of glucosinolates (e.g., homomethionine and aldoximes) that may represses phenylpropanoid production [34].

#### 2.1.2. The Main Effect of the Macrocation Composition on Leaf Metabolome Is Wide

To understand the commonalities and specificities accounted for by the different macrocations’ ratios, we looked at the simple effect of each NS. The post hoc one-way analysis of the metabolites’ accumulation indicated that, by grouping the genotypes, 794 compounds were differentially accumulating (FDR < 0.05) (Supplementary Table S2). The SK solution caused the higher variations, considering both the number of compounds that specifically accumulated in higher (*n* = 267) or lower (*n* = 144) quantities as compared with the other two treatments (Table 1).

**Figure 5:**
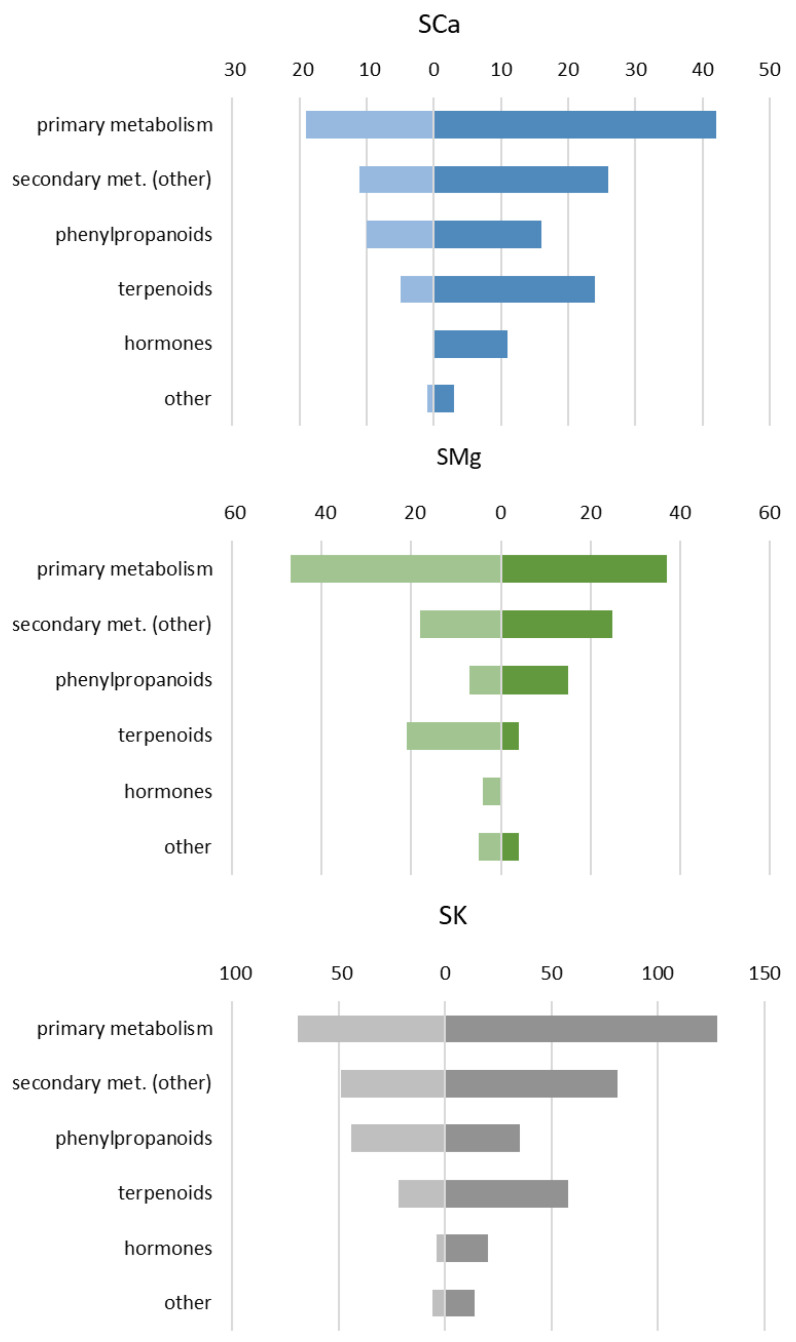
Distribution of the differentially accumulating leaf metabolites from plants growing with the different NSs (SCa, SMg, and SCa). For each class of metabolites, the bars display the number of statistically different compounds (*p* < 0.05, one-way ANOVA) that are present in higher (darker color) and lower (lighter color) amounts. Metabolites and their classification are reported in Supplementary Table S3.

A classification of the compounds that have been altered by each NS relative to the others, regardless of the genotype, is summarized in Figure 5. Considering that the analysis refers to different differently colored varieties, we adopted a less stringent and detailed classification of metabolites, which were ultimately clustered in five main groups (plus “other”).

For all the NSs, the large effect was on primary metabolism, indicating the important role that the macrocations under investigation have in normal leaf growth and development. It should also be added that several metabolites have been included in the category “primary metabolism”, such as nucleotides and derivatives, amino acids and derivatives, (non-sterol) fatty acids, and organic acids and derivatives. Compared to the solution with a dominance of calcium (SCa) and potassium (SK), the SMg solution was the only one associated with a larger number of underrepresented compounds involved in primary metabolism, suggesting that the increased amount of magnesium cannot counteract the reduction of calcium and potassium. Larger differences among the effect of the NSs were present for the secondary metabolites, particularly terpenoids. The ratio between over- and underrepresented terpenoids was higher for SCa solution as compared with SK solution, while the ratio was negative for SMg solution. There was a statistically different atom frequency between the under- (36.4 carbons) and over- (14.2 carbons) accumulating terpenoids for the SCa solution (Supplementary Figure S1). Overall, the SCa solution mostly increased sesquiterpene lactones and other shorter terpenes, while it decreased (linear) tetraterpenoids. In addition, the SCa and SMg solutions (and not the SK solution) presented a positive ratio between over- and underrepresented polyphenols. Among polyphenols, the SMg solution mostly accumulated compounds related to the flavonol biosynthesis, while the SCa soluiton to the flavonoids. The SCa solution also had a large effect on hormones. Specifically, jasmonates (JAs) and more generally, oxylipins were mostly overrepresented. Among the “other secondary metabolites”, compounds related to the biosynthesis of fatty acids and other lipids, including sterols from isoprenoid precursors, were typically present in higher quantities in the SK soluiton, along with chlorophyll and two other metabolites related to porphyrin metabolism. The SK solution had the biggest impact on alkaloids, with 21 compounds significantly altered by this NS (16 up, 5 down) out of the 30 differentially expressed considering the three NSs.

#### 2.1.3. The Metabolic Response of the Varieties to the NSs Is Largely Genotype Specific

**Figure 6:**
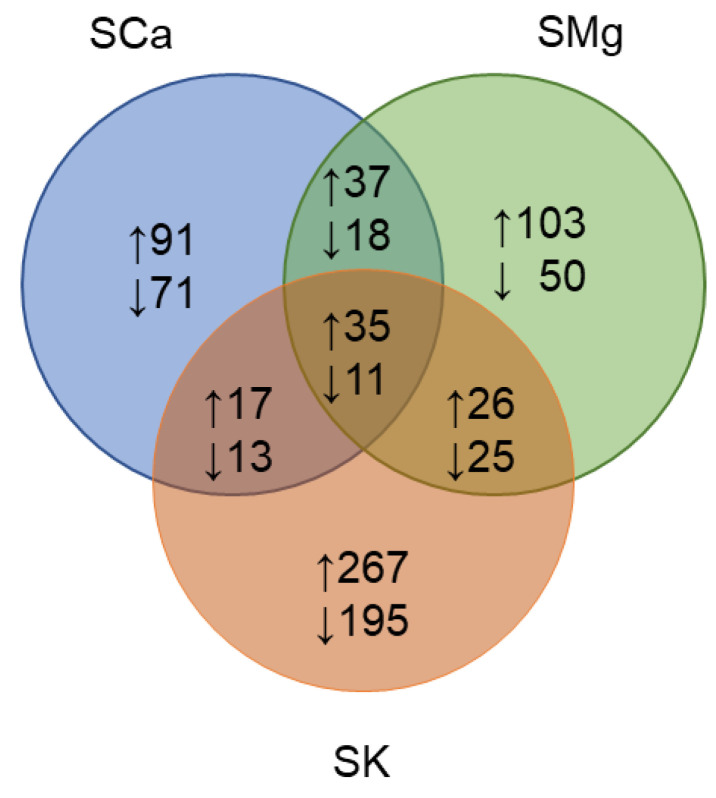
Venn diagram of the differentially accumulating metabolites between the ‘Red Salanova’ and the ‘Green Salanova’ growing in the different nutrient solutions (SCa, SMg, and SK). The graph reports the number of common and specific differentially accumulating compounds. Compounds in higher (respectively, lower) quantities are denoted by an upwards (respectively, downwards) arrow.

After the analysis of the between-group differences, we performed post hoc tests of pairwise contrasts to compare the specific treatments in relation to the genotype under investigation. Our aim was to understand if the different nutrient solutions could specifically modify the pre-existing metabolomic differences between the red-pigmented and the green-pigmented cultivar. The set of differentially accumulating metabolites in the two varieties that were common in two or all NSs were always limited (Figure 6).

For instance, the number of metabolites exclusively altered by each NS (i.e., 162 for the SCa, 153 for the SMg, and 492 for the SK) was always the higher of the number of metabolites differentially accumulating in more than one condition (numbers are reported in the intersecting areas of Figure 6). All this indicated that each variety possesses a specific metabolic profile in each nutrient solution. Moreover, in each NS, the ‘Red Salanova’ generally had a higher number of genotype-specific compounds. Therefore, it is not incidental that, among the 33 compounds accumulating in higher quantities in all conditions, there were 21 flavonoids (including five anthocyanins such as cyanidin-glucoside), and two anthraquinone derivatives, a class of colored compounds. Other potential chromogenic compounds were one glucoside derivate and one flavan. Among the 11 commonly under accumulated metabolites, the amino-acid group (*n* = 4) was the only enriched class of compounds.

**Figure 7:**
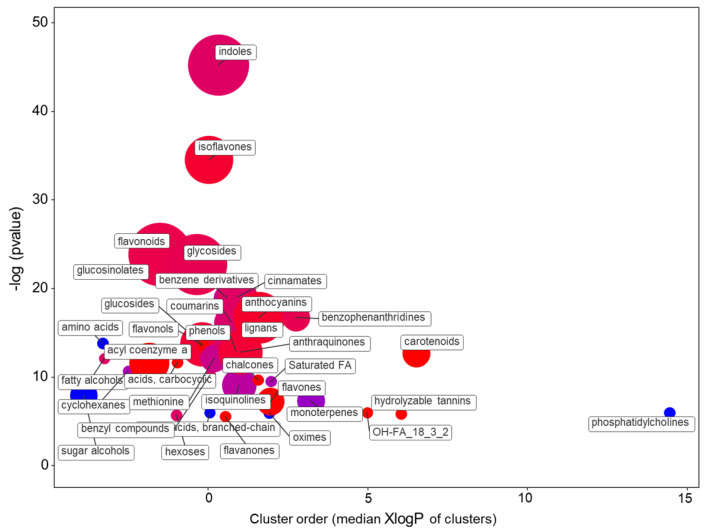
Enrichment statistics plot for the SCa nutrient solution. Each circle indicates a significantly altered cluster of metabolites. The enrichment *p*-values (y-scale) were calculated with the non-parametric Kolmogorov–Smirnov (KS) test. The diameter of the circle is proportionate to the number of metabolites in each cluster. The color scale (from red to blue) illustrates the ratio between increased and decreased compounds in ‘Red Salanova’ as compared with ‘Green Salanova’.

The comparison of the leaf metabolomes of the ‘Red Salanova’ and ‘Green Salanova’ fed with the SCa solution indicated that 694 compounds had a |log2FC|> 1 (425 increased and 269 decreased) (Supplementary Table S4). The *t*-test analysis with correction for multiple comparisons yielded 437 compounds with an FDR < 0.05. The statistically differentially accumulating metabolites were analyzed and classified according to chemical ontologies and structural similarities (Figure 7 and Supplementary Table S5).

The enrichment statistics indicated that the most significantly altered group of metabolites was related to indoles (eight up, four down). In this cluster were included compounds related to the indole-3-acetic acid conjugate biosynthesis, as well as indole alkaloids derived biosynthesis from tryptophan. Among phenylpropanoids, the largest impact was on isoflavones (seven up and one down), followed by flavonoids (ten up and three down), and anthoxanthins (i.e., flavonols, flavones, and flavanones, for a total of ten up and zero down). In addition, the flavonoid precursors chalcones (three up, zero down) and anthocyanins (eight up and one down) were significantly overrepresented in the ‘Red Salanova’. Other affected phenylpropanoids were various lignols and coumarins (three up and two down). Four carotenoids were accumulated in the red genotype while monoterpens were reduced.

**Figure 8:**
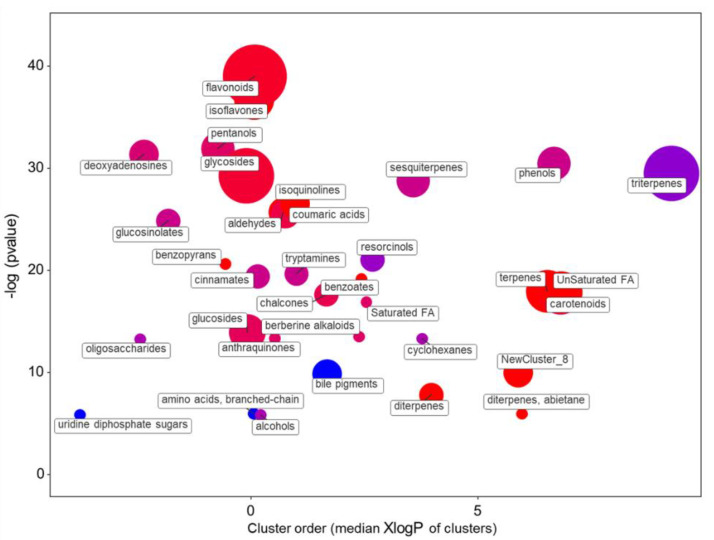
Enrichment statistics plot for the SMg nutrient solution. Each circle indicates a significantly altered cluster of metabolites. The enrichment *p*-values (y-scale) were calculated with the non-parametric KS test. The diameter of the circle is proportionate to the number of metabolites in each cluster. The color scale (from red to blue) illustrates the ratio between increased and decreased compounds in ‘Red Salanova’ as compared with ‘Green Salanova’.

The comparison of the leaf metabolomes of the varieties growing in the SMg solution indicated that a similar number of compounds (581) as compared with SCa were affected, having a |log2FC|> 1 (Supplementary Table S4). A ratio very similar to the SCa was also present considering the up- (356) and the underaccumulated (225) compounds. The *t*-test analysis with correction for multiple comparisons yielded 495 compounds. Nonetheless, the chemical enrichment analysis indicated that the SMg solution causes various metabolic differences between the ‘Red Salanova’ and ‘Green Salanova’ (Figure 8 and Supplementary Table S6).

The most significantly altered groups of metabolites were flavonoids (18 up and two down) and isoflavones (eight up and zero down). Large differences were present for the isoprenoids. Triterpenes were strongly under accumulated (three up and 12 down), while terpenes (nine up and zero down), carotenoids (six up and three down) and ditepernes (four up and zero down) were in higher relative quantities. Similarly, compounds clustered as being related to diterpenoid biosynthesis (i.e., “diterpenes, abietane, and the NewCluster_8”, mainly constituted by abitane-type diterpenes) [35] were also increased.

**Figure 9:**
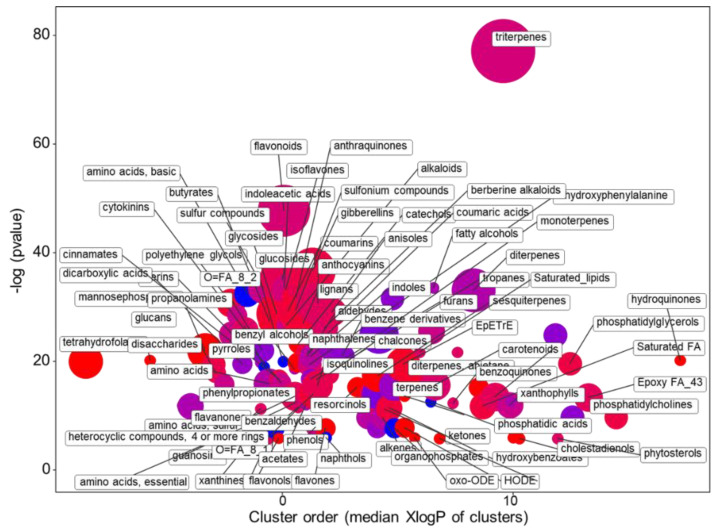
Enrichment statistics plot for the SK nutrient solution. Each circle indicates a significantly altered cluster of metabolites. The enrichment *p*-values (y-scale) were calculated with the non-parametric KS test. The diameter of the circle is proportionate to the number of metabolites in each cluster. The color scale (from red to blue) illustrates the ratio between increased and decreased compounds in ‘Red Salanova’ as compared with ‘Green Salanova’. To increase readability, not all circles (e.g., the purple ones) are annotated.

The statistical analysis of the metabolic profile of the ‘Red Salanova’ and ‘Green Salanova’ supplemented with the SK solution revealed that 916 compounds were differentially accumulated (FDR < 0.05). In total, 677 compounds (respectively, 117) had a log2 FC higher than 1 (resp., lower than −1) (Supplementary Table S4), indicating that the metabolic difference between the genotypes was mostly enhanced by the potassium-enriched NS. As expected, considering the number of compounds, the chemical enrichment analysis showed the presence of several groups of structurally and ontologically related metabolites (Figure 9 and Supplementary Table S7). The most significant impact was present for triterpenes (23 up and 17 down), followed by flavonoids (15 up and ten down). Among isoprenoids, sesquiterpenes were more abundant (nine up and zero down), while diterpenes (six up and 11 down) and monoterpenes (two up and 11 down) were mostly reduced. Only for the SK solution were present differences in the plant hormones, such as gibberellins (15 up and four down) and cytokinins (four up and two down). Moreover, among the compounds classified as “indoleacetic acids” there were two overexpressed auxins (CID: 91820526; 74706).

## 3. Discussion

While recent genomics and transcriptomics initiatives are consolidating lettuce as a model system for Asteraceae and leafy greens [27,36], relatively little is known on the abundance and diversity of the chemical profiles among and within the various horticultural groups [37]. Under this perspective, multifactorial nutritional experiments provide relevant knowledge because mineral elements are one of the most important preharvest factors to shape the phytochemical and functional properties of the edible product. Moreover, the way crops adapt to mineral availability is of fundamental importance to understand the plant mechanisms of nutrient acquisition and adaptation.

Our work highlighted the vast metabolomic diversity in varieties belonging to the same horticultural class (i.e., butterhead lettuce), but it also indicated that such assortment can be heavily manipulated by macrocation availability. The overall biochemical range of compounds is consistent with previously published works on various types of lettuce [38,39,40]. Although the comparison of experimental outcomes may be restricted by non-identical technical features, our work putatively identified a larger number of compounds, and this is likely due to the analysis of metabolic different cultivars and also because of multiple growing conditions [41,42]. The range of altered metabolites is nutritionally significant, because it included vitamins and other phytochemicals with known antioxidant attributes in lettuce, mainly polyphenolics such as anthocyanins and flavonoids (e.g., quercetin, kaempferol, and luteolin derivatives) [43]. These quality compounds, differently from sucrose and ascorbic acid, vary little during post-harvest cold storage [44].

Both factors tested and their interaction significantly impacted the metabolomic profile of the leaves. To gain broad insights into the lettuce biological system, we first performed a functional over-representation analysis using the Arabidopsis background reference set. The enrichment of the compound specifically altered by the genotype factor indicated that two major groups of secondary compounds with functions in the molecular interaction with the environment were impacted (i.e., non-structural phenolics and alkaloids). Consistently, pathways involving the amino acids phenylalanine, tryptophan, and tyrosine, which represent major points of contact between primary and secondary metabolism in plants, were significantly altered. Specifically, compounds deriving from these aromatic amino acids include most of phenolics, indole-derivatives (including alkaloids), as well as anthocyanin pigments. The data suggests that a relevant part of the metabolic difference at the basis of the different coloration of the ‘Red Salanova’ and ‘Green Salanova’ is under robust genetic control and it is little altered by the growing conditions. Taking into account the number of compounds, the NS was the factor with the larger effect, consistent with the concurrent manipulation of three macrocations in the NSs. Interestingly, the NS factor most significantly impacted the carotenoid pathways, as well as the terpenoid backbone biosynthesis (e.g., isoprenoids) among secondary metabolites. The macrocation proportion had a strong influence also on primary metabolism, including the accumulation of proline and related glutamate compounds. The glutamate pathway for proline biosynthesis has been connected to stress [45], in particular salt and heavy metals, but this explanation can be ruled out considering the use of isosmotic solutions and the moderate effect of the NS factor on the leaf water content.

The post hoc analysis of the simple effect of the different nutrient solutions indicated a modest similarity of changes of the metabolite pools. The large array of altered metabolites involved in primary and secondary metabolism due to an increased potassium concentration should depend on its vital role for a range of cellular functions. A positive correlation between alkaloid accumulation and potassium concentration in leaves has been previously reported [46], although, more often, it has been reported that a significant depletion of K usually resulted in a decrease in their total amount [47]. However, a nutrient-specific hallmark in the metabolite level or type, which may result from the strong dysregulation of a metabolic pathway, was not present, probably coherent with the use of nutrient solutions with altered, but not limiting, amounts of mineral elements. Overall, the statistical grouping of the differentially accumulating compounds more closely resembled a multivariate pattern of accumulation, rather than the occurrence of linear response to a single component [48]. Nonetheless, some NS-dependent specific effects were also evident, such as the accumulation of different class of isoprenoids. Moreover, the solution with the highest concentration of calcium induced the accumulation of compounds belonging to the oxylipin-jasmonic acid pathway, including jasmonates. In addition to its essential role for normal plant growth and physiology, calcium is generally seen as a second messenger in stress response [49]. Plants have several calcium-dependent protein sensors typically activated by a transient increase in intracellular calcium levels [50]. In lettuce, an elevated level of oxylipins is associated with a faster post-harvest browning [38] and, in the future, it would be of interest to address whether the constitutive overaccumulation of oxylipins and jasmonates with the SCa solution influences this post-harvest quality factor.

Our current understanding of how the controlled manipulation of multiple mineral elements interacts on a pre-existing phytochemical diversity in plants is very limited, also because, for instance, different studies have been carried out in relation to an exposure to abiotic stress conditions [51,52,53,54]. The analysis of the effects on the NS in relation to the different colored genotypes provided interesting insights. Firstly, the metabolomics’ differences between the green and the red cultivar are largely shaped by macrocation ratios, a result that underlines the biochemical plasticity of lettuce, as well as the potential of obtaining, and plausibly enhancing, distinct functional profiles of the edible product also within the same horticultural group of lettuce [37]. Briefly, different NSs specifically altered the profile of structural (e.g., lignols, SCa) and non-structural phenylpropanoids (e.g., flavonoids, SMg and isoflavonoids, SCa), as well as compound that can affect palatability and the dietary phytonutrient profile (e.g., coumarins, SCa; alkaloids, SCa and SK; triterpenes, SMg and SK; different carotenoids, SMg, SCa, and SK). The SK solution caused the large amplification of the difference between the varieties, consistent with the overall large effect of potassium on the lettuce biochemical profile. Secondly, it is interesting that the set of NS-independent differences between cultivars is limited and highly characterized by putatively chromogenic compounds. This result implies that lettuce breeding for leaf color has selected and fixed a relatively reduced, but highly stable, biochemical trait [55].

## 4. Materials and Methods

### 4.1. Plant Material and Experimental Design

We studied two lettuce (*Lactuca sativa* L. var. capitata) multi-leaf butterhead varieties, ‘Descartes’ RZ (also known as ‘Green Salanova’) and ‘Klee’ RZ (also known as ‘Red Salanova’), both from Rijk Zwaan Italia (Bologna, Italy). Plants grew in an open-gas-exchange growth chamber equipped with high pressure sodium lamps, under a photosynthetic photon flux density of 420 μmol/m^2^/s and with 12:12 h photoperiod. During the light period (resp. dark) temperature was set at 24 °C (18 °C) and relative humidity at 65% (75%). Plants grew in hydroponics using the Nutrient Film Technique (NFT) with a 15.5 plants/m^2^ density, as previously reported [32]. We employed a randomized complete-block design with three replicates, for a total of 18 experimental units. Each experimental unit was made of one NFT channel with 12 plants, of which the first and last two were considered to be guard plants. The two experimental factors were the varieties (two levels) and the nutrient solution (NS), with three levels namely, a high proportion of potassium (SK), of calcium (SCa), and of magnesium (SMg). The concentrations of the three varying cations were as reported [32], for a cationic ratio of 0.68 K/0.16 Ca/0.16 Mg for the SK solution, of 0.16 K/0.68 Ca/0.16 Mg for the SCa solution, and of 0.16 K/0.16 Ca/0.68 Mg for the SMg solution. The concentration of the other mineral elements was constant in all the NSs [32], for a total macronutrient concentration of 24 meq/L. The electrical conductivity and the pH were monitored and adjusted if necessary, within the 1.5 ± 0.1 dS/m and 6.0 ± 0.1 intervals, respectively.

### 4.2. Analysis of Leaf Traits

Leaf characteristics such as the leaf average biomass (LAB) and the leaf average area (LAA) were measured at harvest (19 days after transplant), as previously described [32]. The specific leaf area (SLA) was computed as SLA = (total leaf area)/(total leaf dry weight) and expressed in cm^2^/g [56]. Leaf succulence (LSU) was calculated as LSU = (leaf fresh weight—leaf dry weight)/(leaf area) and expressed in mg/cm^2^ [57]. Statistical differences were evaluated by a two-way analysis of variance (ANOVA), followed by a post hoc test (Student’s *t*-test) for pairwise comparison of the two varieties in each nutrient solution. Calculations and visualization were performed in R 4.02.

### 4.3. Metabolomics and Data Analysis

Metabolites’ extraction, chromatography, mass spectrometry, data acquisition, spectral preprocessing, and feature extraction were performed as described [58]. Briefly, leaf samples (1.0 g DW) were extracted in 20 mL of 0.1% formic acid 4:1 (*v*/*v*) methanol/water solution, homogenized, and filtered through a 0.2 µm cellulose disposable syringe cartridge. Small metabolites were analyzed by ultra-performance liquid chromatography (UHPLC)–QTOF mass spectrometry, using a 1290 Infinity UHPLC System and a G6550 iFunnel Q-TOF LC/MS mass spectrometer (Agilent, Santa Clara, CA, USA). Raw data are presented elsewhere [59]. Data filtering was achieved using the interquartile range of the peak intensity. Variable centering was carried out with the autoscaling function (i.e., variables were mean centered and divided by their standard deviation). A two-way ANOVA statistical test was applied to determine the effect of each factor (the genotype, two levels and the nutrient solution, three levels) and their interaction. The statistical significance (*p*-value) was adjusted for multiple comparisons (false discovery rate (FDR) based on the Benjamini–Hochberg procedure). The pathway enrichment analysis and pathway topology analysis of the main effect of the factors (the genotype, the nutrient solution, and their interaction) were performed retrieving the KEGG codes associated with the statistically different compounds of the two-way ANOVA. This over representation analysis, based on the hypergeometric test, was performed considering the reference *Arabidopsis thaliana* pathway library (Oct. 2019), and adjusting the *p*-values for multiple comparisons (Fisher’s FDR). The post hoc exploratory analysis of the main effect of the nutrient solution was carried out using a univariate analysis of variance (one-way ANOVA, FDR < 0.05), while differences between the two genotypes in each nutrient solution were evaluated using an independent Student’s *t*-test. These analyses, along with principal component analysis, hierarchical clustering, and heatmap visualization were carried out with Metaboanalyst 4.0 [60]. The classification of the differential compounds into biochemical classes was manually curated following the information retrieved from the PubChem [61] and the MetaCyc [62] database. For the chemical enrichment analysis, compounds were grouped considering chemical ontologies and structural similarity integrated with the fold change and the statistical significance (corrected *t*-test *p*-values) [63].

## 5. Conclusions

Our work highlighted the biochemical plasticity of lettuce to multifactorial variation of mineral availability and it provided evidence and insights regarding the intraspecific metabolic adaptation in response to macrocation proportions. Breeding for different colors ensured a specific subset of differentiating compounds, and the two lettuce varieties have separate metabolic strategies, in terms of abundance of biochemical classes, to respond to environmental variation. In the future, it will be interesting to assess the contribution of genetic factors to the here described intraspecific metabolic variation [55], as well as the functional contribution of the different chemical profiles to quality-related traits in lettuce, in order to translate this knowledge into new lettuce lines with an improved quality and enhanced functional benefits.

## Figures and Tables

**Table 1:** A summary of the statistical grouping of the differentially accumulating compounds. The different NSs (SCa, SMg, and SK) were compared using one-way ANOVA. The table reports the number of compounds (*n*) that followed the various statistical homogenous groups (FDR < 0.05). In each row, the letter “a” denotes the highest mean value, followed by “b” and “c” (the lowest).

SCa	SMg	SK	*n*
a	a	b	144
a	ab	b	22
a	b	a	73
a	b	ab	9
a	b	b	93
a	b	c	13
a	c	b	16
ab	a	b	5
ab	b	a	18
b	a	a	36
b	a	ab	1
b	a	b	44
b	a	c	37
b	ab	a	14
b	b	a	245
b	c	a	14
c	a	b	4
c	b	a	6

## Data Availability

The data presented in this study are openly available in the MDPI Data repository, at 10.3390/data5040119.

## Supplementary Materials

Supplementary materials can be found at https://www.mdpi.com/2223-7747/10/1/91/s1, Figure S1: Box and whisker plots of the carbon atom frequency in the differentially accumulated terpenoids identified in our study. For each nutrient solution (SCa, SMg, SK), the graph displays the median (thick black line) and the outliers (black dots) for the underrepresented (down) and overrepresented (up) terpenoids. For each pairwise comparison, the statistical significance (Student’s *t*-test) was for SCa, *p* = 0.0073; for SMg, *p* = 0.056; and for SK, *p* = 0.96, Table S1: Two-way ANOVA summary table of the variables under investigation. See main text for the abbreviations. Significance code *** *p* < 0.001, ** *p* < 0.01, * *p* < 0.05, and ns *p* > 0.05, Table S2: Summary table of the one-way ANOVA of the metabolites considering the main effect of the nutrient solutions (SCa, SMg, and SK), Table S3: Annotation of the statistically different metabolites considering the main effect of the nutrient solution (one-way ANOVA), Table S4: List of the compounds differentially accumulating (|log2FC|> 1) between the ‘Red’ and ‘Green Salanova’ in the three NSs (Sca, SMg, and SK), Table S5: Summary statistics of the chemical enrichment analysis between the ‘Red’ and ‘Green Salanova’ in the SCa solution, Table S6: Summary statistics of the chemical enrichment analysis between the ‘Red’ and ‘Green Salanova’ in the SMg solution, Table S7: Summary statistics of the chemical enrichment analysis between the ‘Red’ and ‘Green Salanova’ in the SK solution.
