# Supplementary material for: Intraspecific Variability Largely Affects the Leaf Metabolomics Response to Isosmotic Macrocation Variations in Two Divergent Lettuce (Lactuca sativa L.) Varieties
Source: Plants (Basel). 2021 Jan 5;10(1):91. doi: 10.3390/plants10010091 (PMC7824788; doi:10.3390/plants10010091)
Supplement: Supplementary file 1 [file plants-10-00091-s001.zip › Supplementary Table 1.docx]

Supplementary Table 1: Two-way ANOVA summary table of the variables under investigation. See main text for the abbreviations. Significance code: *** p<0.001; ** p<0.01; * p< 0.05; ns p> 0.05.

| **Variable** | **Factor** | **Df** | **Sum Sq** | **Mean Sq** | **F value** | **p value** | **significance** |
| --- | --- | --- | --- | --- | --- | --- | --- |
| LAB | Genotype | 1 | 0.00691 | 0.006911 | 4.028 | 0.06782 | ns |
|  | NS | 2 | 0.04252 | 0.021262 | 12.393 | 0.00121 | ** |
|  | Genotype x NS | 2 | 0.04128 | 0.020642 | 12.032 | 0.00136 | ** |
|  | Residuals | 12 | 0.02059 | 0.001716 |  |  |  |
| LAA | Genotype | 1 | 24.693 | 24.693 | 88.633 | 6.85E-07 | *** |
|  | NS | 2 | 1.399 | 0.7 | 2.511 | 0.12273 | ns |
|  | Genotype x NS | 2 | 9.485 | 4.742 | 17.023 | 0.000313 | *** |
|  | Residuals | 12 | 3.343 | 0.279 |  |  |  |
| SLA | Genotype | 1 | 5185 | 5185 | 24.775 | 0.000321 | *** |
|  | NS | 2 | 1156 | 578 | 2.761 | 0.103152 | ns |
|  | Genotype x NS | 2 | 294 | 147 | 0.703 | 0.514229 | ns |
|  | Residuals | 12 | 2511 | 209 |  |  |  |
| LSU | Genotype | 1 | 125.83 | 125.83 | 32.717 | 9.61E-05 | *** |
|  | NS | 2 | 47.87 | 23.93 | 6.223 | 0.014 | * |
|  | Genotype x NS | 2 | 2.89 | 1.45 | 0.376 | 0.694 | ns |
|  | Residuals | 12 | 46.15 | 3.85 |  |  |  |
