# Supplementary figures and images for: Intraspecific Variability Largely Affects the Leaf Metabolomics Response to Isosmotic Macrocation Variations in Two Divergent Lettuce (Lactuca sativa L.) Varieties
Source: Plants (Basel). 2021 Jan 5;10(1):91. doi: 10.3390/plants10010091 (PMC7824788; doi:10.3390/plants10010091)

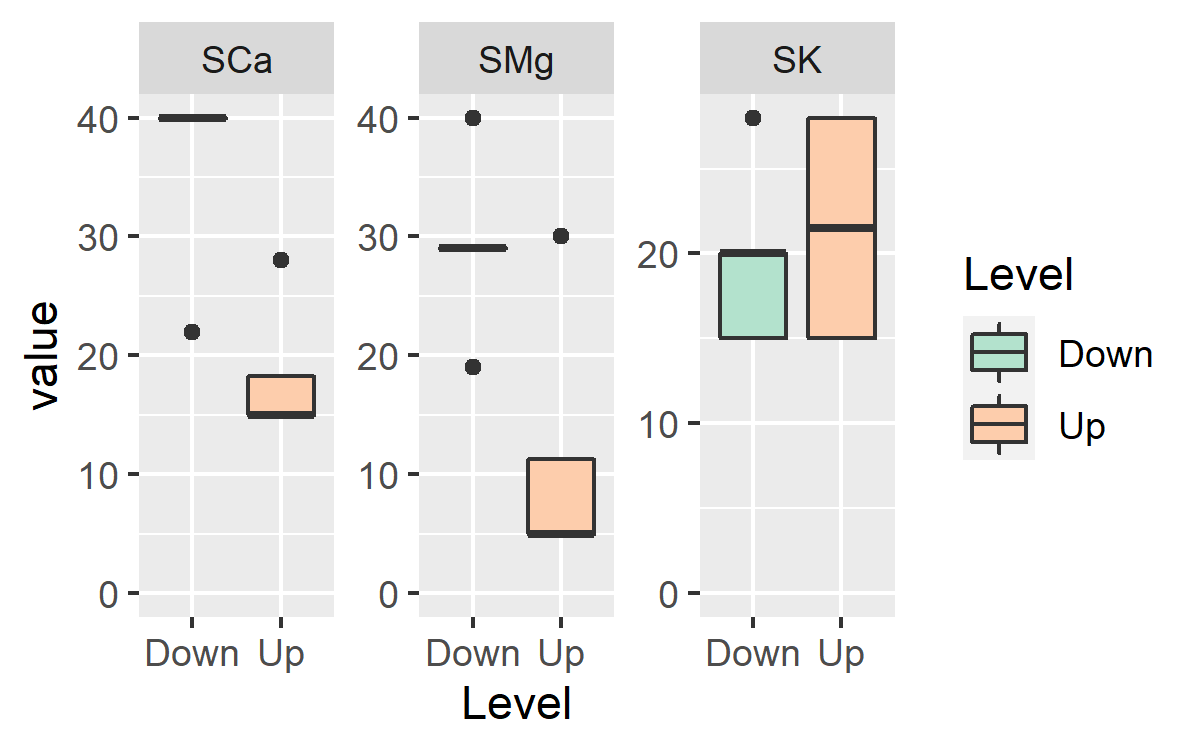

Supplement: Supplementary file 1 [file plants-10-00091-s001.zip › Supplementary Figure 1.docx]
